# An Adaptive Brightness Global Digital Image Correlation Method for Deformation Measurement Using Overexposed Images

**DOI:** 10.3390/s25133957

**Published:** 2025-06-25

**Authors:** Chunyuan Gong, Boxing Qian, Qianhai Lu

**Affiliations:** 1 State Key Laboratory for Manufacturing Systems Engineering, School of Mechanical Engineering, Xi’an Jiao Tong University, Xi’an 710049, China; 2 School of Mechanical and Precision Instrument Engineering, Xi’an University of Technology, Xi’an 710048, China; qianboxing1023@126.com (B.Q.); 1210211009@stu.xaut.edu.cn (Q.L.); 3 School of Mechanical Engineering, Xian Aeronautical Institute, Xi’an 710077, China

**Keywords:** brightness restoration, finite element partitioning, global digital image correlation, deformation measurement

## Abstract

In deformation measurements, processing overexposed images poses challenges due to the welding process or metal reflection. To track the deformation surface, an Adaptive Brightness Global Digital Image Correlation method is proposed. First, the effective range is determined based on the extent of image overexposure. Second, an improved Dark Channel Prior method is employed to adjust the brightness of overexposed images. Third, by calculating the parameter results of Finite Element Partitioning, Adaptive Brightness Global Digital Image Correlation can be utilized to conduct deformation measurements. The proposed method can adjust both the image brightness and Finite Element Partitioning for Global Digital Image Correlation. The experimental results demonstrate that the improved dark channel method modifies the image brightness without altering its brightness distribution. The modified image can significantly increase the Mean Intensity Gradient within different partitions. This method overcomes the difficulty in measuring the weld deformation during the welding process and can achieve dynamic deformation measurement using overexposed images. Finally, the evolution processes of unstable deformation and angular deformation in the whole welding field are obtained, which can assist in optimizing the welding process.

## 1. Introduction

The deformation measurement during the welding process plays a vital role in welding parameter analysis. It is of great significance for accurately simulating the welding process and optimizing the welding procedure. T-joint welding finds extensive applications, and research on its welding simulation mainly focuses on deformation, temperature field, and residual stress field distribution. Peng, Jin et al. [1] conducted a numerical simulation of the laser welding process of T-joints to explore the impact of bilateral asynchronous and bilateral synchronous laser welding on molten pool stability. They also analyzed the influence of welding sequences on post-weld equivalent stress and deformation in three stringers’ T-joints. Ding, Hong Tao et al. [2] used the numerical simulation method to study both the magnitude and distribution of the temperature field, residual stress, and deformation of DH36 steel fillet welded T-joints. The Finite Element simulation results show good agreement with experimental measurements in terms of temperature distribution, melt pool morphology, residual stress, and deformation distribution. The study indicates that welding speed and power are crucial for ensuring welding quality. Ding, Hong Tao et al. [3] investigated the influence of welding sequences and boundary conditions in welding. It was found that tightening the ends of the flange plate with bolts during the welding process can significantly reduce the deformation. Wu, Han Ling et al. [4] studied the welding deformation of T-joint ribs under multi-point constraint conditions through numerical simulation and experimental methods, demonstrating that the multi-point constraint method can effectively control angular deformation. However, at present, only the deformation measurements of certain key points are employed to validate the welding process. Although Digital Image Correlation (DIC) methods are widely employed in deformation measurement, they encounter issues due to changes in image brightness, such as overexposure that occurs during the measurement of welding deformation or reflection from metal surfaces.

When measuring the deformation of T-joint welding, image overexposure occurs, which makes it challenging to acquire deformation information near the weld joints. Owing to the lack of deformation measurement data in the vicinity of the T-joints, numerical simulations lack comparative verification data. Without the means to compare and analyze the measurement results, the optimization of welding process parameters yields limited effects. This verification method has limited data and lacks the correlation between weld deformation and full-field instability deformation. During the T-joint welding process, three major problems arise. First, the weld area features over-saturated regions, resulting in the loss of grayscale information in the image. Second, uneven brightness variations in the image lead to a decrease in the correlation of stereoscopic images, which can render DIC methods ineffective. Third, in T-joint welding, the rib plate and base material are located at different spatial positions, i.e., not on the same plane, making it difficult to obtain the weld deformation of T-joint using conventional DIC methods.

In order to address the issues of weak correlation caused by image brightness, such as the aforementioned welding radiation and metal reflection, some scholars have conducted research on image enhancement for measurements. The main focus of those researchers is to perform image correction for issues like image atomization interference, underexposure, or overexposure. He, Ming Kai et al. [5] proposed a Dark Channel Prior Algorithm to remove haze from a single input image. This algorithm can directly estimate the haze thickness and restore a high-quality haze-free image. Hou, Guo Jia et al. [6] developed an illumination channel sparse prior guided variational framework for the restoration of non-uniformly illuminated underwater images. Zhou, Jing Chun et al. [7] proposed an underwater image recovery method, which effectively alleviates problems caused by solid-colored objects and highlighted regions in underwater scenarios. Rinanto, Noorman et al. [8] proposed a system consisting of two different residual attention convolution networks. These networks are responsible for restoring the lighting conditions of degraded images by utilizing a residual memory block network with a self-attention layer. However, the above-mentioned image enhancement algorithms encounter difficulties when processing welding images. Since the dark channel information of patterns in welding images incorporates both radiation light and background information, direct segmentation and restoration are challenging. Additionally, there is a loss of grayscale in the overexposed area of welding images, which leads to a reduction in image correlation.

Due to the limitation of missing grayscale data in overexposed images, it is difficult to directly obtain the full-field deformation process of the weld using conventional Digital Image Correlation (DIC) methods. A Global/Finite Element Digital Image Correlation (Global DIC or Finite Element DIC) method [9] is employed to estimate displacement fields from pairs of images. This method has garnered widespread attention in the academic community and has achieved remarkable advancements in related research [10,11,12], demonstrating its potential and superiority in the field of deformation measurement. Van, Beeck et al. [13] applied the Global DIC method with linear triangular Finite Element to track the three-dimensional material movement based on the measured height profiles. Ma, Shao Peng et al. [14] proposed an eight-node quadrangular mesh-based DIC method. For the measurement of a heterogeneous deformation field, the eight-node quadrangular mesh Digital Image Correlation method exhibits higher accuracy. Wang, Bo et al. [15] pointed out several important considerations in the practical implementation of the FE-based DIC algorithm. The results indicate that the improved FE-based DIC method has obvious advantages in terms of accuracy and efficiency. Kleinendorst, S.M. et al. [16] proposed a Mechanical Shape Correlation method when the speckle pattern degrades due to large or complex deformations. In Mechanical Shape Correlation (MSC), the images are projections based solely on the contour lines of the test specimen. Wang, X. et al. [17] proposed non-uniform eight-node quadrilateral (Q8) elements to process displacement fields with regions of both high and low gradient. The results show that when the shape function of an element matches or over-matches the gradient of the displacement field, a larger element size can yield better results. Wittevrongel, L. et al. [18] proposed an adaptive Global DIC method. The algorithm focuses on accurately determining small strains with high strain gradients. The self-adaptive principle reduces the dependence of the results on the user’s input, and the higher orders ensure sufficient degrees of freedom. The Global DIC method, constrained by the grid topology, can measure the discontinuous deformation field and obtain its dynamic full-field deformation [19,20]. Tao, Rao et al. [21] described the application of the global conforming Finite Element-based DIC technique to obtain precise kinematic fields at the interfaces between dissimilar materials. This technique can achieve a high level of accuracy for the interfacial region of composites or joints. The above-mentioned methods for measuring discontinuous deformation fields can be categorized into two types. One is to add degrees of freedom within the Finite Element, which can enhance the flexibility of the model but may increase computational complexity. The other is to align the Finite Element boundary with the boundary of the discontinuous deformation field to ensure continuous deformation within. This approach simplifies the calculation process and is thus chosen later because it is easier to implement. Sciuti, V. F. et al. [22] analyzed the localized and diffuse sources of brightness changes during the implementation of Global DIC. Brightness and contrast corrections can be beneficial to the registration procedure. However, the correction of overexposed images has not yet been applied in the study of Global DIC measurement.

This study innovatively proposes an Adaptive Brightness Global Digital Image Correlation method for image brightness restoration, which is a significant improvement in the field of welding deformation measurement. This method achieves image overexposure brightness adjustment based on an improved Dark Channel Prior method, mitigates the influence of brightness variations on Global Digital Image Correlation, and adaptively adjusts the Finite Element Partitioning according to the effectiveness parameters. This new approach enables the dynamic measurement of welds and full-field deformation during T-joint welding, providing a more efficient and reliable solution for welding quality control.

## 2. Materials and Methods

The proposed method is an improvement of the Dark Channel Prior Algorithm and the Global Digital Image Correlation method. A brief introduction to these two methods will be given.

### 2.1. Dark Channel Prior Algorithm

The Dark Channel Prior Algorithm is often used to extract and repair the effects of ambient light, which has gained significant popularity in the field of image processing. The brightness of the image can be expressed as shown in Equation (1):

(1) $$I(x,y)=J_{0}^{A}(x,y)(1-V(x,y)/B_{\infty })+V(x,y)$$

where *J*_0_*^A^*(*x*,*y*) represents the original image, *V*(*x*,*y*) represents the intensity of the ambient light, and *B_∞_* represents the backscatter in a line of sight which extends to infinity.

First, calculate the dark channel intensity of the image, as shown in Equation (2):

(2) $$J^{dark}(x,y)=\min(I^{c}(x,y))$$

where *I^c^* is the color channel of the image.

Then, in order to eliminate the influence of random noise, median filtering is employed here, and it can preserve significant jumps along edges simultaneously. The intensity of the ambient light can be obtained by median filtering, as shown in Equation (3):

(3) $$V(x,y)=median_{s}(J^{dark}(x,y))$$

where *S* represents the size of the filtering window, and it can be selected according to the average size of the object particles in the image.

In Equation (1), *B_∞_* can be obtained from the brightest intensity within the background light intensity. It is generally considered to be among the top 1% of the brightest pixels.

Based on the current model, normal image restoration can be achieved as shown in Equation (4):

(4) $$J_{0}^{A}(x,y)=\frac{I(x,y)-V(x,y)}{1-V(x,y)/B_{\infty }}$$

### 2.2. Global Digital Image Correlation Method

**Figure 1:**
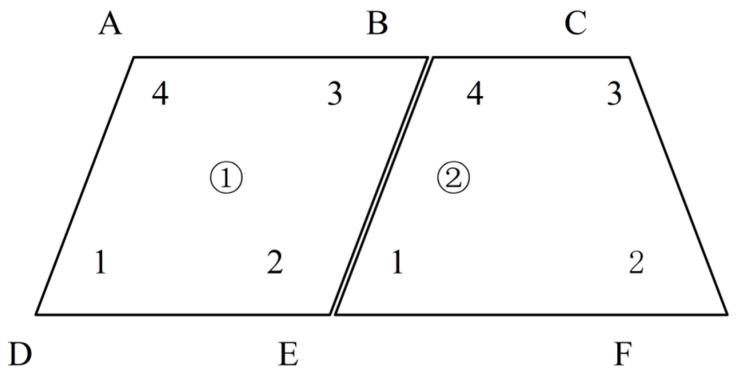
The schematic diagram of the topological relationship in the Global Digital Image Correlation Method.

The Global Digital Image Correlation method is capable of conducting an overall match of the image. This is because the displacement of Finite Element nodes is computed by the comprehensive grayscale information within the whole field. The topological relationship among the Finite Elements guarantees the uniqueness of each matching point, as illustrated in Figure 1.

The two elements have the common boundary points B and E. In the first element, node 3 and node 2 coincide with node 4 and node 1 in the second element. In this way, the Global Digital Image Correlation (GDIC) can integrate the two elements, thereby obtaining the overall deformation field.

When utilizing the Global DIC method for correlation matching, the primary step is to partition the Finite Element in accordance with the procedures of traditional Finite Element simulation. Subsequently, the node positions of each element are correlated to obtain the deformation of the entire field. The objective function used in the Global DIC method [9] is the sum of the squares of the grayscale, as shown in Equation (5):

(5) $$\eta^{2}={\iint_{\Omega }\left[u(x)\cdot \nabla f(x)+f(x)-g(x)\right]}^{2}dx$$

where the images of the reference and deformed states are, respectively, called *f*(*x*) and *g*(*x*); *u*(*x*) represents the displacement field; and ∇*f*(*x*) represents the gradient of *f*(*x*).

## 3. An Adaptive Global Digital Image Correlation Method Using Overexposed Images

To solve the problem of image supersaturation during welding, this paper conducts brightness restoration for the high-temperature radiation. The image can be divided into two regions according to its characteristics. One is the area where the brightness generated by high-temperature radiation in the image exceeds the measurement range; this area is defined as the overexposed area. The other is the area significantly affected by the radiation, usually the region close to the radiation source, which is referred to as the affected area in this paper. The overexposed area, where grayscale information loss occurs and causes image matching failure, should be clearly marked. Subsequently, brightness restoration is performed on the speckled characteristic information within the affected area. These different situations need to be dealt with separately to achieve adaptive correction. Finally, to track the whole-field deformation, the Finite Elements are partitioned according to the size of the overexposed area. In summary, the process of adaptively correcting overexposed images can be divided into the following three steps: (1) segmentation of the overexposed areas to precisely identify regions with information loss; (2) restoration of brightness in the affected area, enhancing the contrast of the speckled characteristic information; (3) partitioning of Finite Element based on the overexposed area and performing Global Digital Image Correlation calculations to obtain accurate whole-field deformation results.

### 3.1. Segmentation of the Overexposed Area

To eliminate the supersaturated components in the image, the first step is to reduce the influence of high-frequency noise points. Initially, both the original image and the deformed image are filtered to uniformly eliminate the high-frequency noise within the images. Subsequently, the overexposed area is searched for and marked. In the deformed image, the grayscale values of the overexposed area are close to or equal to 255. Therefore, the areas in the grayscale information with values greater than a certain threshold are directly marked. The threshold can be set as 245. In the subsequent calculations, the marked overexposed area can be applied to the selection of the deformation image matching range and also to the restoration of the image brightness.

(6) $$\left\{\begin{array}{c} H(x,y)=1,G(x,y)>g_{0}\\ H(x,y)=0.G(x,y)\leq g_{0} \end{array}\right.$$

where *g*_0_ is 245. As the image is first filtered to remove high-frequency noise, the brightness of the original overexposed area decreases, and 245 is selected as the threshold.

### 3.2. Improved Dark Channel Prior Algorithm

The grayscale information of the image changes in the vicinity of the weld radiation source. This leads to weak correlation in the obtained image, making it difficult to perform calculations. To address the issue of image restoration, it can be resolved by improving the Dark Channel Prior Algorithm.

For grayscale images, the initial value of the dark channel in Formula (2) is the grayscale image itself. However, the initial value not only contains the light source radiation information but also the background brightness information of the speckle image. Directly restoring this image will change the overall brightness of the image. Thus, further extraction of the information within the dark channel is necessary. To eliminate the background brightness of speckle brightness, the overexposed area range has been used to modify the Dark Channel Prior Algorithm.

The affected area is closely adjacent to the overexposed area, and the farther the distance from the overexposed area in the image, the smaller the influence. Therefore, perform Gaussian filtering on the overexposed area, and use the filtering result as the scope of the dark channel in the affected area.

The filtering result can be expressed as shown in Equation (7):

(7) $$A(x,y)={gauss}_{s_{2}}(H(x,y))$$

where *s*_2_ represents the size of the Gaussian filtering. The average size of the overexposed area in the image is taken as the filtering radius.

To achieve the normalized superposition of the affected areas of the Dark Channel Prior Algorithm, the following correction parameters can be expressed as Equation (8):

(8) $$B(x,y)=A(x,y)\cdot \frac{1-H(x,y)}{h_{0}}$$

where *h*_0_ represents the maximum value in the affected area.

In this manner, by combining the correction parameters *B*(*x*,*y*) with the intensity of the ambient light *V*(*x*,*y*), the modified intensity of the ambient light can be expressed as shown in Equation (9):

(9) $$V(x,y{)}^{\prime }=B(x,y)\cdot V(x,y)$$

Image restoration can be accomplished by substituting this modified intensity into the relevant Formula (4).

### 3.3. Adaptive Finite Element Partitioning

Firstly, the initial Finite Element is partitioned for the left camera image, thereby obtaining the initial value of the reference displacement of the left camera. It should be noted that when measuring the deformation fields of different materials or with different spatial arrangements, the element boundaries need to be fixed to pass through the contact areas of materials or spatial contact points. For example, when measuring T-joint welding, the element boundaries must align with the weld path. Then, taking the left camera as the reference, the Zero Normalized Sum of Squared Differences (ZNSSD) is employed to determine the initial value of the undeformed reference displacement in the right camera. Thirdly, the invalid area is removed for the adaptive restoration of the image. It is necessary to determine whether there is image oversaturation caused by welding. The highlight oversaturated area is removed, and the images of the surrounding area are corrected for matching. Fourthly, the Finite Element size is adjusted adaptively, and the effectiveness of the Finite Element is judged by the following three judgment criteria. To dynamically select the element size, various sizes (e.g., ranging from 5 × 5 to 120 × 120 pixels) are tested. First, the minimum element size is tested. If the selection criteria are not met, the size is increased, and the validation continues. When the smallest element size that satisfies all three criteria is found, the adaptive partitioning process is completed. According to the above steps, all states are calculated through the matching calculation, and the deformation of the T-joint welding is obtained.

The three judgment criteria for the adaptive partitioning of Finite Element sizes are shown as follows.

#### 3.3.1. Mean Intensity Gradient

After determining the measurement area, the Finite Element can be partitioned. To avoid the situation where an overly small interval size leads to insufficient internal information and makes matching difficult, a reasonable element size needs to be selected. This ensures that each Element contains sufficient grayscale gradient information. Here, the Mean Intensity Gradient is used to evaluate the grayscale gradient information, and it can be expressed as Equation (10):

(10) $$MIG=\frac{1}{W\times H}\sum_{x=1}^{W}\sum_{y=1}^{H}\sqrt{f_{x}{\left(x_{i},y_{i}\right)}^{2}+f_{y}{\left(x_{i},y_{i}\right)}^{2}}$$

where *W* and *H* represent the width and height of each element, respectively, and *f_x_* and *f_y_* represent the grayscale gradients in the X/Y direction inside each element.

#### 3.3.2. Average Speckle Integrity in the Element

The average speckle integrity is used to evaluate the relationship between the size of the speckle and the size of the element. If the size of the element is too small, it will result in uneven speckle information within the element. This can be expressed as Equation (11):

(11) $$C=\frac{N_{Comp}}{N_{All}}$$

where *N_Comp_* represents the number of speckles completely inside the element, and *N_All_* represents the sum of all speckles that can be observed in the element.

#### 3.3.3. Effective Grayscale Percentage

The effective grayscale percentage is used to evaluate the relationship between the size of the overexposed area and the size of the element. After the initial image is partitioned into Finite Elements, due to the removal of the overexposed area, the amount of grayscale information within the Finite Elements is reduced. The literature has discussed the node displacement of the elements for Global DICs in detail. It can be seen that almost all grayscale information in the elements has an impact on the displacement of the nodes. Therefore, in this article, we try to use part of the grayscale information to match the Global DIC. Here, a validity determination criterion is added when performing Global DIC matching to ensure the accuracy of the matching. Effective grayscale percentage is the ratio of the effective grayscale points in the element to the number of measurement points in the element. It can be expressed as Equation (12):

(12) $$T(i,j)=\frac{\sum k_{ij}}{n_{ij}}$$

where *k_ij_* represents the number of pixels at which the brightness of the element is valid, and *n_ij_* represents the total number of pixels in the element. To ensure the validity of the measurement results, the threshold value can be set as 70%.

When we perform adaptive Finite Element partitioning, we utilize the above three parameters to characterize the quality of the image speckle. Different element sizes significantly influence both the Mean Intensity Gradient and speckle integrity within the element. Specifically, a larger average grayscale gradient and higher speckle integrity within the element indicate that the grayscale characteristics of the element are more distinct. We employ the third parameter to determine whether the valid information in the image meets the specified validity threshold. If not, it means that the grayscale information used in the Global Digital Image Correlation calculation is insufficient, which leads to inaccurate displacement data. Therefore, the element size needs to be adjusted. In this way, the accuracy of the Global DIC calculation results can be guaranteed.

## 4. Experiment

In the experiment, the experimental conditions were first introduced. Then, the restoration effect of the proposed improved dark channel method was discussed. Next, the parameters for the adaptive partitioning of the Finite Element were discussed. Finally, the results of the T-joint welding were analyzed.

### 4.1. Experimental Conditions

**Figure 2:**
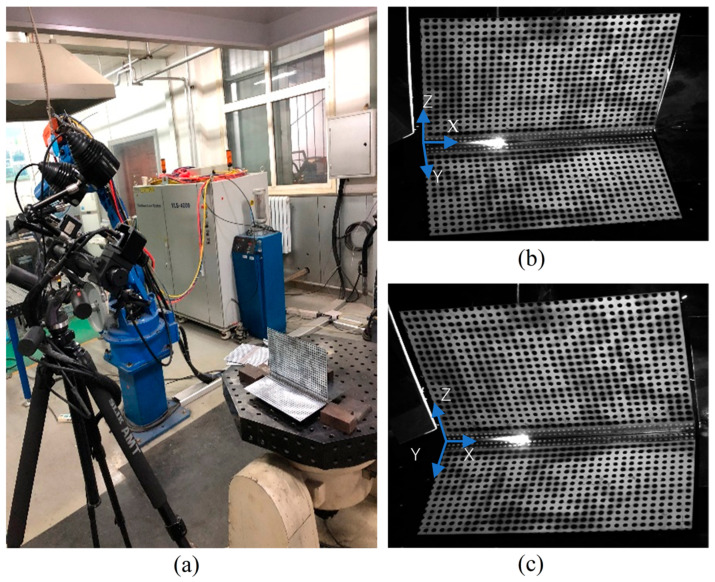
Experimental scene diagram of the welding scenario. (**a**) Data acquisition equipment for welding processing; (**b**) image from left camera; (**c**) image from right camera.

During the welding process, the plate material is BS700MC (Shanghai, China), with dimensions of 300 mm × 300 mm × 2 mm. The material of the reinforcement rib is Q235 (Shanghai, China), and its dimensions are 300 mm × 200 mm × 2 mm. The welding is conducted using gas metal arc welding. The specific welding parameters are as follows: the welding current is 150 A, the voltage is 17 V, the welding speed is 8 mm/s, and the flow rate of the protective gas is 15 L/min. First, spot welding is carried out on the plate. Then the welding deformation test is performed, as shown in Figure 2.

The specific data acquisition parameters are as follows: the resolution of the camera is 1920 × 1200 pixels, the focal length of the lens is 23 mm, the distance between the camera and the object is approximately 90 cm, and the calibration accuracy is 0.02 pixels. Figure 2a shows the data acquisition equipment for the welding process. Examples of the images captured by the camera at a certain moment during the experiment are shown in Figure 2b,c. It can be observed that this speckle pattern is superimposed with radiation light, resulting in issues such as weak correlation and difficulty in matching.

### 4.2. Image Brightness Restoration Effect

**Figure 3:**
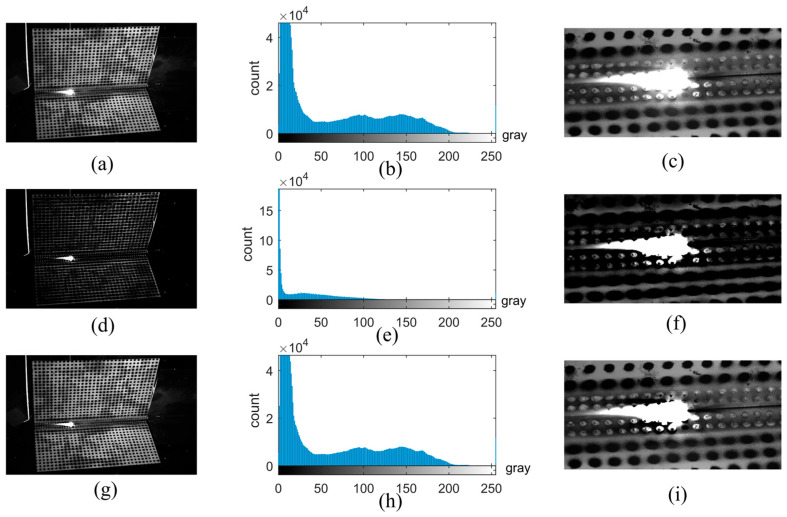
The effect of the Improved Dark Channel Prior Algorithm. (**a**–**c**) The original image, its grayscale distribution, and a locally magnified view; (**d**–**f**) the restored image by the Dark Channel Prior method, its grayscale distribution, and a locally magnified view; (**g**–**i**) the restored image by the proposed method, its grayscale distribution, and a locally magnified view.

Images were collected during the welding operation. The objectives of the image brightness restoration are as follows: (1) Divide the overexposed area and mark it. (2) Restore the image’s brightness distribution and contrast. The images collected during welding can be processed by applying the Improved Dark Channel Prior Algorithm. To illustrate the effect of image restoration, the original image, the image processed by the Dark Channel Prior Algorithm, and the proposed method are all shown in Figure 3.

As shown in Figure 3, the proposed image restoration method can achieve a grayscale distribution similar to the original image, whereas the grayscale distribution of the conventional Dark Channel Prior method is significantly altered. It is difficult to distinguish the radiation light and speckle background by directly using the Dark Channel Prior method. In contrast, the proposed method enables the brightness of the unsaturated area to change uniformly and maintain a similar overall grayscale distribution, thus demonstrating a superior restoration effect.

### 4.3. The Finite Element Partitioning in Global DIC

In the welding measurement experiments, the size of the speckle remains unchanged, and the radius of the molten pool changes only slightly. Therefore, it is assumed that the radius of the overexposed area in the image is relatively constant. By analyzing the parameters of any image, appropriate parameters for Finite Element partitioning can be selected and applied to the entire welding process.

**Figure 4:**
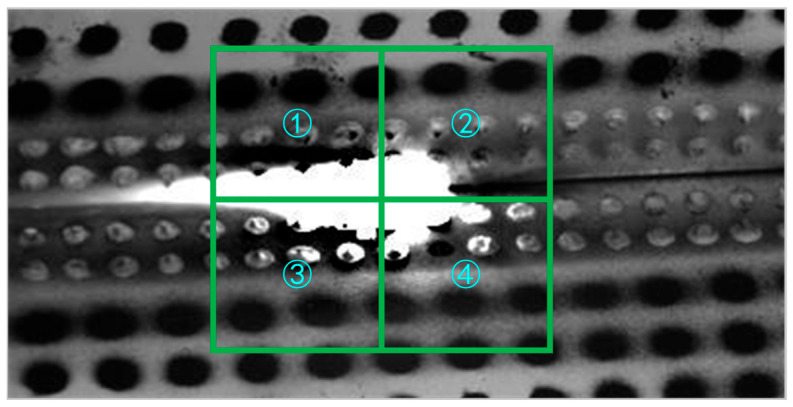
Finite Element Partitioning for the restored image.

**Figure 5:**
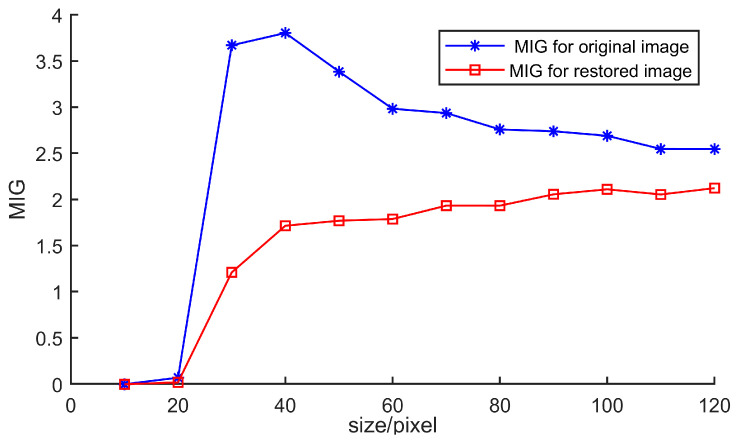
The Mean Intensity Gradient for the original image and the restored image.

**Figure 6:**
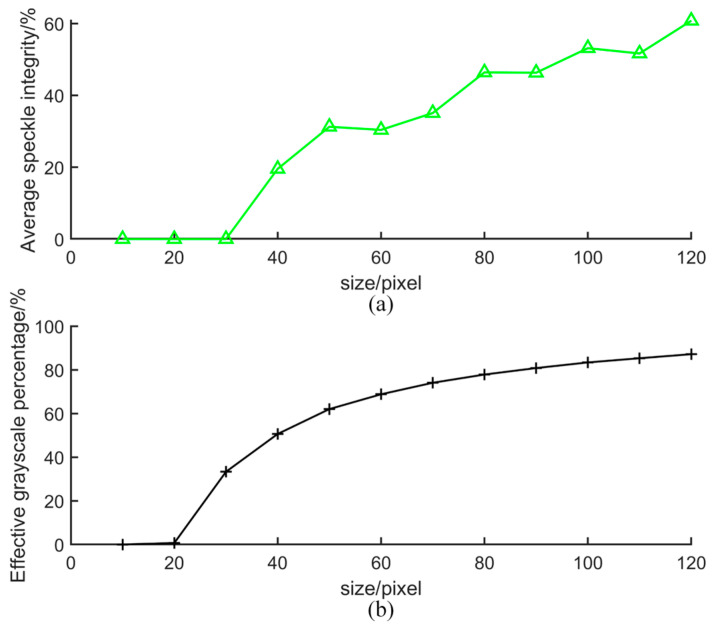
Average speckle integrity in the element and the effective grayscale percentage for the restored image. (**a**) Average speckle integrity in the element for the restored image; (**b**) the effective grayscale percentage for the restored image.

The size of the Finite Element partitioning can be calculated for the restored image by the following steps. Firstly, the center of the molten pool is selected as a fixed point, and the Global DIC is partitioned into four elements of random sizes as shown in Figure 4. To dynamically select the size of the Finite Element, a series of sizes from 5 × 5 pixels to 120 × 120 pixels is changed for testing. According to the aforementioned three judgment criteria, the results for different sizes are presented in Figure 5 and Figure 6.

Corresponding to different Finite Element partitioning sizes, the Mean Intensity Gradient before and after image restoration can be computed, as shown in Figure 5. The Mean Intensity Gradient is larger for the restored image than for the original image, which indicates that the image restoration effect is remarkable. For the restored image, it can be observed that when the average grayscale gradient is less than 20 pixels, the average grayscale value is relatively low. This phenomenon reflects the insufficiency of grayscale information in the welding molten pool part, leading to a decrease in the Mean Intensity Gradient. When the Finite Element partitioning size ranges from 20 to 50 pixels, the Mean Intensity Gradient is slightly higher. This is because the gradient between the highlight information of the welding molten pool and the surrounding grayscale information is large. When the size exceeds 60 pixels, the Mean Intensity Gradient remains stable. Therefore, based on the Mean Intensity Gradient analysis, to ensure reliable results, the Finite Element partitioning size should be larger than 60 pixels.

Both the average speckle integrity in the element and the effective grayscale percentage are consistent for the original image and the restored image. The two judgment criteria for a series of Finite Element partitioning sizes are calculated, as shown in Figure 6. When the Finite Element partitioning size is less than 30 pixels, there are scarcely any complete speckles within the element. This is due to the fact that the characteristics of the welding molten pool lead to the loss of speckle grayscale information. However, when the Finite Element partitioning size ranges from 30 to 70 pixels, average speckle integrity shows little change. This is because the size is close to the average speckle size in the image. When the size exceeds 80 pixels, the speckle integrity within the element is significantly enhanced. This is because the number of complete speckles contained in the element increases. When the size reaches 100 pixels, the percentage of speckle integrity exceeds 50%. Therefore, according to the speckle integrity, a size of 100 pixels can be chosen. When the Finite Element partitioning size is greater than 70 pixels, the effective grayscale percentage is relatively stable. Thus, based on the above three criteria, a Finite Element size of 100 pixels is selected for the full-field deformation analysis.

### 4.4. The Results of the Proposed Method and XTDIC

**Figure 7:**
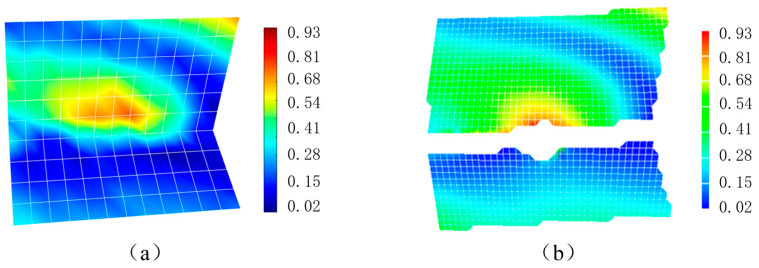
Comparison of deformation fields obtained by two algorithms. (**a**) Deformation field of the proposed method; (**b**) deformation field of XTDIC.

As a commercial software, the measurement accuracy of XTDIC (Version 8.3.2) can be guaranteed. To illustrate the accuracy of the proposed method, an average displacement field obtained by the proposed method and the XTDIC software at an arbitrary moment (t = 17 s) during the welding process is selected for verification, as shown in Figure 7.

It can be seen that both the proposed algorithm and the DIC algorithm can obtain the full-field deformation. The full-field displacements measured by the two algorithms are consistent in magnitude and direction. However, the DIC algorithm itself cannot measure the spatial discontinuous deformation boundary, so discontinuous points are generated near the weld. Therefore, the DIC method loses the deformation information near the weld, while the AFE-DIC method makes the weld boundary consistent with the elements boundary, ensuring that all used data participate in the calculation to obtain the deformation field, and can obtain the deformation process near the T-joint.

### 4.5. Welding Full Field Deformation

**Figure 8:**
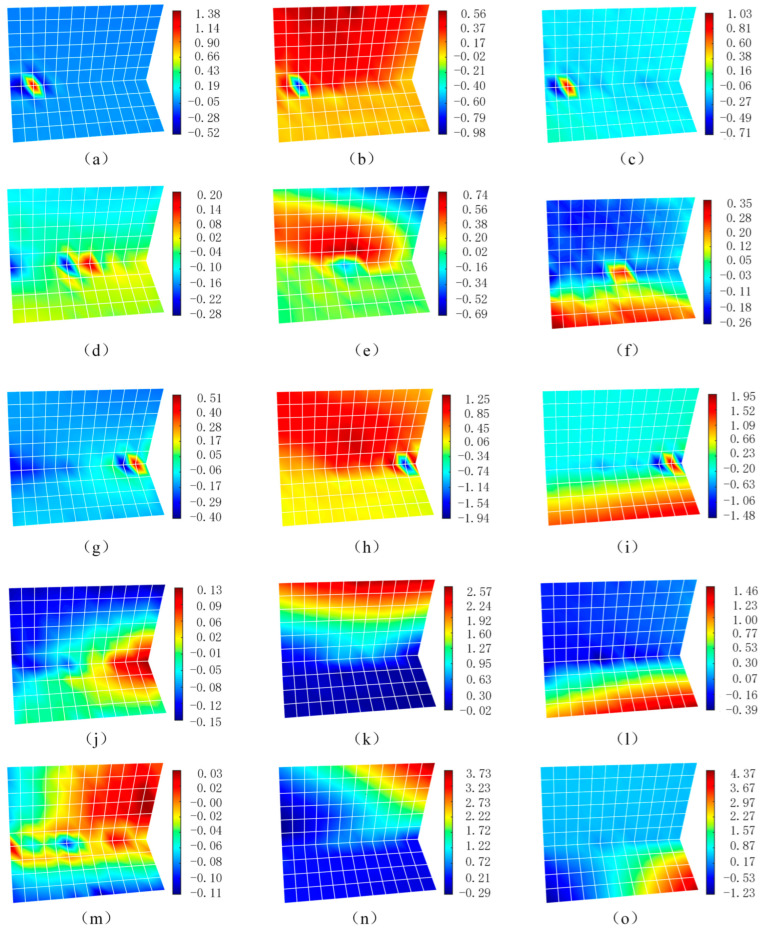
Full-field deformation evolution process during welding. (**a**–**c**) The deformations in the X, Y, and Z directions at the beginning of welding (*t* = 2 s); (**d**–**f**) the deformations in the X, Y, and Z directions in the middle stage of the welding (*t* = 17 s); (**g**–**i**) the deformations in the X, Y, and Z directions when the welding arc is extinguished (*t* = 32 s); (**j**–**l**) the deformations in the X, Y, and Z directions during the cooling process (*t* = 67 s); (**m**–**o**) the deformations in the X, Y, and Z directions at the end of the cooling (*t* = 157 s).

During the welding process, the welding gun moves at a constant speed for welding. To illustrate the evolution of deformation throughout the welding process, five specific moments are selected: the start of welding (*t* = 2 s), the middle stage of welding (*t* = 17 s), the moment of arc extinguishing during welding (*t* = 32 s), the cooling stage (*t* = 67 s), and the end of the cooling stage (*t* = 157 s). According to the algorithm proposed in this article, the full-field deformation diagram of the entire welding process is obtained, as shown in Figure 8. The coordinate direction is the same as that depicted in Figure 2.

It can be observed that at the beginning of welding, the temperature at the weld is the highest, causing the nearby metal to expand due to heat. Since the temperature of the surrounding metal is lower than that of the weld area, compressive stress is generated on the weld area. At this moment, it is clearly evident that the material near the weld point has undergone displacement. The weld point moves 1.38 mm and −0.52 mm in the two sides of the X direction, respectively, and moves −0.98 mm in the Y direction. The rib plate moves 0.56 mm in the Y direction, and the two sides of the weld point move 1.03 mm and −0.71 mm in the Z direction, respectively. This indicates that at the start of welding, the expansion of the metal leads to the displacement of the peripheral material, and the whole rib plate moves in the positive Y direction, resulting in a decrease in the angle between the rib plate and base material.

When the welding reaches the middle stage, it can be seen that the lateral displacement on the left side of the weld point shows a trend of moving leftward on the left side and rightward on the right side. This suggests that during the welding process, the material near the weld expands. At this time, the deformation in the Y direction is still greater than that in the X direction, which is similar to the situation at the start of welding. The difference is that at the beginning of welding, the overall displacement and deformation of the rib plate are relatively small, while, when the welding progresses to the middle stage, the rib plate exhibits obvious angular deformation. At this moment, the angle between the rib plate and the base material increases, and the base material also bends upward, showing angular deformation.

By the end of the welding, the deformation amount on the left side of the weld becomes −0.4 mm. At this time, the lateral expansion of the weld reaches its maximum value. Through analyzing the Y-direction deformation field and the Z-direction deformation field, it can be found that at the end of the welding, the amounts of the entire deformation are relatively uniform, and the angular deformation becomes more pronounced, with the angle between the rib plate and base material decreasing. This is because the heat conduction during welding raises the overall temperature. Meanwhile, the weld area remains at a relatively high temperature, making the overall deformation of the rib plate and base material more likely to occur, thus resulting in obvious angular deformation.

It can be seen from the displacement change in the X direction after welding that although the displacement of the weld area during the cooling process (*t* = 65 s) is smaller compared to the time when the welding is completed, obvious lateral expansion can still be observed, and it does not turn into shrinkage after welding (*t* = 155 s). After welding, during the cooling process, it can be observed that the angle is further reduced, and the angular deformation on the right side gradually develops due to uniform shrinkage along the weld (*t* = 155 s). It can be concluded that during the cooling process, the weld shrinks, the tensile stress gradually increases, and the compressive stress gradually decreases. This is manifested as the weld deforming with stress changes, which causes the rib plate and base material to undergo out-of-plane deformation and obvious angular deformation.

## 5. Discussion

During the welding process, both the welding arc light and the radiation light from the welding pool cause the photosensitive units on the camera to become saturated, resulting in white images at the corresponding positions of the photosensitive elements. This leads to large-area overexposure near the welding zone, making it impossible to identify the image information around the welding joints. Traditional methods of observing the area near the molten pool with a narrowband filter suffer from the problem of difficult full-field deformation measurement, as the image brightness on the surface of the rib plate and base material is too dark to perform Digital Image Correlation calculations. During the measurement process, full-field deformation measurement requires grayscale information on the surface of the test piece. Therefore, the following two measures are adopted to improve the grayscale information on the surface of the test piece: (1) the welding side and the measurement side are in different directions to avoid the influence of welding arc light as much as possible; (2) a polarizing filter is used to increase the contrast of the image. Implementing the above measures can effectively improve the overall grayscale information of the image, but the overexposed area near the molten pool still remains.

In terms of image brightness processing, the method in this paper improves the dark channel method by incorporating the overexposed area to achieve adaptive adjustment of image brightness. The traditional dark channel method achieves correction by selecting the darkest part of the RGB image as prior information. During welding deformation measurement, since speckle images are applied on the surfaces of the rib plate and base material, if the light and dark changes in the speckle images are mistakenly treated as part of the Dark Channel Prior information, the brightness of the speckle images will alter after correction, which leads to errors in speckle matching and hinders Digital Image Correlation calculation. As shown in Section 4.3, the grayscale changes in the speckle images significantly affect the grayscale distribution. Therefore, direct application of the dark channel method for image restoration is ineffective.

When selecting adaptive partitioning parameters, one of the images is randomly selected for testing. After determining the size, the same parameters are used for full-field calculation. For adaptive brightness restoration, the image adaptive restoration is performed according to the effective range of each image. In fact, the images are captured by a high-speed camera, and the proposed method is processed offline. This method can be applied to other welding scenarios, but it is necessary to ensure that the element boundary aligns with the weld path. For example, when welding two materials, the element boundary should be fixed on the T-joint. Otherwise, the element containing both materials will cause calculation errors.

In DIC experiments, MIG is commonly used for image evaluation—the higher the MIG, the more conducive it is to the implementation of DIC. This method aims to address the situation where the images of the rib plate and base material surface are clear, but overexposure occurs at the welding joint. The image brightness restoration method proposed here can significantly increase the MIG value. In the experiment, parameters such as welding speed must be optimized to obtain properly exposed images. The overexposed images of the surfaces lead to a decrease in MIG and make it difficult to calculate DIC results. Underexposure, meanwhile, may render only the molten pool morphology observable while darkening the welding image surfaces, also reducing MIG and ultimately hindering full-field deformation detection.

This paper uses commercial XTDIC software to verify the effectiveness of the proposed method. The results show that the overall trends are consistent. Compared with XTDIC, this method significantly expands the measurement range, which is conducive to the analysis of welding instability deformation and the optimization of welding processes.

## 6. Conclusions

The proposed method can effectively address the issue of speckle matching failure caused by image brightness changes. It can effectively track the displacement at the T-joint, enabling the weakly correlated matching of the deformation in discontinuous areas. Consequently, it is capable of obtaining the full-field welding deformation of T-joint welding.

The Adaptive Brightness Global Digital Image Correlation method described in this paper is manifested in two aspects. Firstly, the T-joint welding image has a high brightness intensity, resulting in image oversaturation. Through image enhancement, it eliminates the influence of the radiation light. Secondly, the adaptability is reflected in the selection of a reasonable Finite Element size to ensure accurate matching. This ensures the effectiveness of the Global DIC measurement, enabling the measurement of large deformations during the welding process.

Through the measurement of the deformation at the T-joint welding, it is found that at the beginning of welding, it first expands outward and subsequently shrinks. The initial welding position experiences a relatively large displacement. When the welding progresses to the middle, the angular deformation of the rib plate is unstable. The displacement in the Y direction changes from positive to negative and then back to positive, which is manifested as a change in the angle between the rib plate and base material. At the end of welding, due to the high temperature, the weld material continues to expand towards the starting point. Ultimately, during the cooling process at the end of welding, the position where welding starts deforms more, while the position where welding ends deforms less. Based on the obtained full-field deformation diagram, the lateral, longitudinal, and angular deformations of the weld are analyzed, providing valuable data for in-depth research on the welding process.

## Data Availability

The data presented in this study are available on request from the corresponding author.

## Abbreviations

The following abbreviations are used in this manuscript:

DIC	Digital Image Correlation
Global DIC	Global Digital Image Correlation
FE DIC	Finite Element Digital Image Correlation
MIG	Mean Intensity Gradient
ZNSSD	Zero Normalized Sum of Squared Differences
IDCPA	Improved Dark Channel Prior Algorithm
